# Optimizing wind-PV-battery microgrids for sustainable and resilient residential communities

**DOI:** 10.1038/s41598-025-06354-6

**Published:** 2025-07-08

**Authors:** Jyotismita Mishra, Ajay Shankar

**Affiliations:** 1 Vellore Institute of Technology, Chennai, 600127 India; 2 Energy Exemplar, Pune, 411028 India

**Keywords:** Energy and society, Sustainability

## Abstract

Integrating solar and wind energy with battery storage systems into microgrids is gaining prominence in both remote areas and high-rise urban buildings. Optimally designing all distributed energy resources (DERs) within a microgrid enhances self-sufficiency, reliability, and economic feasibility. However, due to the inherent unpredictability of DERs, a robust stochastic-based optimization approach is crucial. This article proposes a Grey Wolf-based multi-objective optimization technique for wind-solar-battery-assisted residential microgrids. The method aims to minimize renewable energy costs by determining the optimal sizing of components based on a given microgrid load profile. To address the global energy trilemma, the microgrid is modeled with economic, reliability, and energy indices, ensuring a balanced three-dimensional objective. The proposed algorithm is evaluated across three different configurations, with a numerical analysis of the capacity degradation factor to assess battery lifetime.

## Introduction

Climate change has become a global concern for governments, industries, local bodies, and individuals. Escalating energy demand and an urge to protect the environment have driven a shift towards RESs. This has resulted in a significant increase in the total capacity of wind power generation (WPG) and solar power generation (SPG) facilities throughout the globe^1,2^. However, due to the fluctuating nature of RESs, access to large-scale distributed grids may not be possible for system stability and power quality concerns. Therefore, the microgrid concept has arrived to overcome all these contentions. Moreover, these can be useful for remote area electrification where the power supply from the mainstream grid is nonviable and high-rise urban apartments for stable and sustainable power supply. A microgrid is an integration of distributed renewable energy resources (DRERs), integrated systems with loads, and energy storage devices^3^.

To utilize the DERs effectively and efficiently, it is essential to analyze the microgrid system numerically and develop one optimized model before installation^4–6^. The sizing of the system can be done by many commercial available tools^7–9^, . However, due to limitations like flexibility in objective function, constraint adaptability, large and complex system, handling with stochastic variables, the costumed optimization techniques research are widely popular and gaining attentions. However, because of the microgrid’s complex nature and multiple constraints, the optimal design is difficult by using the classical method. So, many researchers are proposing the optimal sizing of microgrids by enhancing the economic value of the system.

An on-grid renewable energy systems (RESs) optimization technique with multi agent is discussed in^10^. A PV-battery-based techno-economic optimization technique for microgrids is addressed in^11,12^. Another PV-battery-based microgrid is presented in^13^ for optimal size considering the battery life cycle. Though this type of system is economical, the use of only one source may not be reliable for remotely located microgrids. Therefore, the complementary source wind RES can be added to form a hybrid system. With the advancement of different optimization techniques, several research articles presented different sizing techniques for PV-Battery, Wind-Battery^14–16^ and PV-Battery-Diesel^17^, Wind-PV-Battery-Diesel^18^, Pv-wind-battery-fuel cell^19,20^ etc. system configurations. Also in^21^, the monte carlo simulation for uncertainty situation is discussed for wind-diesel hybrid system. Though the Diesel system addition makes it more reliable and autonomous, the environmental adverse effect motivates authors to design one optimized renewable energy-based microgrid for remotely placed locations. In^22^, one simulation-based optimization method is implemented to find the size of the PV panel with the battery. Also, it includes a battery degradation model to find the battery life cycle. A PV-battery-based configuration for household load^23^ is presented to find the optimized results considering the reliability and load increment. Demand response strategies are addressed in the literature^24–27^ for optimal sizing taking into account the market price of the electricity. Stochastic-based pattern searched optimization algorithm is presented in^28,29^. Moreover, many tools are there for optimization of hybrid system such as^7^, Moreover, the short lifetime of the battery with increased capital cost leads the researchers to develop an effective sizing of battery for DERs^30–34^. A two-stage approach optimization technique is proposed for life cycle and optimal size estimation^35^. From various applications, it is noticed that Lithium-ion and Lead-acid batteries are dominant technologies. Due to the low cost, and ruggedness, lead-acid is widely used^36,37^ in India.

For the sizing of DERs, the first step is to identify the location and profile of climatic conditions such as wind speed and solar irradiance. Inefficient capacity leads to an unreliable system. So, the second step for optimization is to model the DERs individually taking into account the extensive load profile and installation area constraints. Simultaneously, one effective multi-objective optimization algorithm is selected for sizing and reliability assessment in which the levelized cost of energy (LCOE), and loss of power supply probability (LPSP) are important indices to evaluate. However, only these indices don’t meet the world energy trilema (WET) index. For the sustainable feature of WET, it is also necessary to minimize the excess energy for the remotely placed microgrid. Furthermore, the energy index is considered as an additional objective function and modeled as a three-dimensional objective to meet the WET index.

A basic and easy-to-implement optimization algorithm is the graphical construction method^38^ in which the number of PV panels with installation area and wind turbine swept area are not considered. This action results in the over/under-sizing of the system. Further, the probabilistic algorithm^39^ also fails to find the optimal solution for dynamic response. A global optimum solution can be effectively identified by using artificial intelligent based algorithm^40–42^ such as Genetic algorithm (GA), Particle swarm optimizer(PSO), ant colony optimization (ACO), artificial bee colony (ABC), harmony search (HS), and cuckoo search (CS). However, it is found that most of the algorithms can’t solve the no-coordinated system and converge in the local optimum point. From the above discussion, it is noticed that an integrative approach-based optimization algorithm is required for the sizing of DERs in microgrids. The contributions of this article are as follows:

1. A meta-heuristic multi-objective grey wolf optimization algorithm is proposed for a wind-solar-battery assisted microgrid system which will be a promising solution for remote locations where the grid connection is nonviable.
2. A detailed mathematical model is developed for the proposed configuration.
3. A three-dimensional objective function is constructed to meet the WET index.
4. Battery lifetime period is evaluated computationally considering the effect of capacity degradation due to corrosion and SOC.

## Modeling of microgrid

**Fig. 1:**
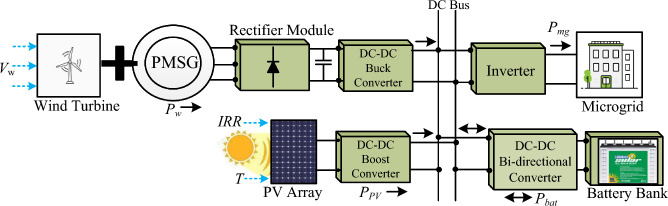
Schematic diagram of Wind-PV-Battery based Microgrid.

A residential microgrid which includes residential houses, small-scale industries, and a few agricultural farms is studied in this article. This microgrid comprising wind, solar, and battery as the major distributed units is shown in Fig. 1. Both the DERs are connected to the DC bus through suitable power electronics converters. A PMSG-based wind generator is interfaced to the DC bus through a 3-$\phi$ rectifier and a boost converter forming the wind power generating unit. The solar power generating unit consists of a PV array and a boost converter. A battery pack is connected with the DC bus via a non-isolated bidirectional converter forming the battery storage unit to smooth the power fluctuations arising due to intermittent behavior of wind and PV power generating unit. Detailed mathematical modeling is crucial for the optimal size of each distributed unit.

### Wind energy generation system model

The power contained in wind ($P_{o}$) is:

1 $$\begin{aligned} {P_\mathrm{{o}}} = \frac{1}{2}\rho AV_\mathrm{{w}}^\mathrm{{3}} \end{aligned}$$

Wind turbine mechanical power ($P_{W}$) is:

2 $$\begin{aligned} P_{W}=\left\{ \begin{matrix} 0, V_{W}<V_{Wcutin}\\ \frac{1}{2}\rho AV_{W}^{3}C_{p}\left( \lambda ,\beta \right) , V_{Wcutin}\le V_{W}\le V_{Wcutout}\\ P_{Wrat}, V_{W}<V_{Wcutout} \end{matrix}\right. \end{aligned}$$

And the tip speed ratio ( $\lambda$ ) is:

3 $$\begin{aligned} \lambda = \frac{{{\omega _\mathrm{{t}}}R}}{{{V_\mathrm{{w}}}}} \end{aligned}$$

Where $\rho$ is the air density, *A* is the rotor blade area, $V_\mathrm{{w}}$ is the wind velocity without rotor interference, $C_\mathrm{{p}}$ is the power co-efficient and $\omega _t$ is the rotor shaft speed of wind turbine, $\beta$ is the pitch angle, $C_{p}$ is a function of $\lambda$ and $\beta$ and is expressed as:

4 $$\begin{aligned} & {C_\mathrm{{p}}}\left( {\lambda ,\beta } \right) = {c_1}\left( {\frac{{{c_2}}}{{{\lambda _i}}} - {c_3}\beta - {c_4}} \right) {e^{ - \frac{{{e_5}}}{\lambda }}} + {c_6}\lambda \end{aligned}$$

5 $$\begin{aligned} & \frac{1}{{{\lambda _\mathrm{{i}}}}} = \frac{1}{{\lambda + 0.08\beta }} - \frac{{0.035}}{{{\beta ^\mathrm{{3}}} + 1}} \end{aligned}$$

Where $$c_{1}$$ = 0.5176, $$c_{2}$$ = 116, $$c_{3}$$= 0.4, $$c_{4}$$ = 5, $$c_{5}$$ = 21, and $$c_{6}$$ = 0.0068.

### Solar energy generation system model

The PV cell is modeled as a current source, diode, and series and parallel connected resistances.

6 $$\begin{aligned} I = {I_{PV}} - {I_D}\left[ {\exp \frac{{q\left( {V + {R_s}I} \right) }}{{akT}} - 1} \right] - \frac{{V + {R_s}I}}{{{R_{sh}}}} \end{aligned}$$

The PV power^22^ is evaluated by using Eq. 7.

7 $$\begin{aligned} P_{PV}=F_{f}V_{PV}I_{PV} \end{aligned}$$

Where $F_f$ is fill factor, $V_{PV}$ and $I_{PV}$ are PV voltage and current respectively.

### Battery storage system model

The battery model is nothing but a combination of dependent voltage source ($E_\mathrm{{bat}}$) and series resistance ($R_\mathrm{{bat}}$)^43^. The corresponding equations are illustrated in (8)–(10). $V_\mathrm{{b}}$ is specified as two different function as $V_\mathrm{{b,ch}}$ and $V_\mathrm{{b,disch}}$.

8 $$\begin{aligned} {V_{\mathrm{{b,ch}}}}= & {v_o} - k\frac{Q}{{Q + 0.1{Q_e}}}{i^{*}} - k\frac{Q}{{Q - {Q_e}}}{Q_e}\nonumber \\ & + La{p^{ - 1}}\left( {\frac{{\exp \left( s \right) }}{{sel\left( s \right) }}\frac{1}{s}} \right) \end{aligned}$$

9 $$\begin{aligned} {V_{\mathrm{{b,disch}}}}= & {V_o} - k\frac{Q}{{Q - {Q_e}}}{i^{*}} - k\frac{Q}{{Q - {Q_e}}}{Q_e}\nonumber \\ & + La{p^{ - 1}}\left( {\frac{{\exp \left( s \right) }}{{sel\left( s \right) }}} \right) \end{aligned}$$

10 $$\begin{aligned} SOC= & \left\{ {1 - \left( {\frac{1}{Q}\int \limits _0^t {{i_{\mathrm{{bat}}}}\left( t \right) dt} } \right) } \right\} \times 100 \end{aligned}$$

Where $v_{0}$ is the voltage constant, *k* is the polarization constant, the maximum capacity of the battery is *Q* is, $Q_\mathrm{{e}}$ is the extracted capacity, $i*$ is the dynamics of low-frequency current, *exp*(*s*) is the dynamics of the exponential zone, *sel*(*s*) is the modes of the battery (0,1 for discharge and charge mode respectively).

#### Energy management strategy

The main aim of the optimization technique is to design a wind-solar-battery-based microgrid system with coordinated energy management strategies (EMS). The EMS is operated based on data received from the power conditioner for wind power, solar power, and battery power concerning the load demand of the microgrid. For this, the total RESs generation ($P_{w}$+$P_{PV}$) SOC is monitored. If the generation exceeds the load power with SOC in a safe range, the battery will be charged and the corresponding mathematical expression is presented in Eq. 12. During this condition, if SOC goes beyond its limit ($SOC_{min}$ $\le$ SOC $\le$ $SOC_{max}$), the EMS should stop the battery and to maintain the power balance, either PV or wind power should be reduced or stop to maintain the power balance.

11 $$\begin{aligned} P_{bat}=[P_{w}+P_{PV}]-P_{L} \end{aligned}$$

Similarly for low generation, the battery power will be delivered to meet the load demand. Again for the low SOC region, the battery controller will be stalled and minimum power is delivered to load.

12 $$\begin{aligned} P_{bat}=P_{L}-[P_{w}+P_{PV}] \end{aligned}$$

#### Aging model

The estimation of battery lifetime is a crucial and complex task for the assessment of reliability evaluation. Here, the Schiffer weighted Ah lead acid battery model^44^ is considered for further evaluation of the remaining capacity ($C_{rem}$) of the battery and this the basic model. In this model, the $C_{rem}$ is calculated by subtracting the capacity loss ($C_{l}$) from the normalized value of initial battery capacity ($C_{n0}$).

13 $$\begin{aligned} C_{rem}=C_{n0}-C_{l} \end{aligned}$$

Further, the loss of battery capacity mainly depends on two essential factors such as battery active mass degradation ($C_{deg}$) and corrosion in positive electrode ($C_{cor}$)^43^. Hence, the capacity loss is the addition of $C_{deg}$ and $C_{cor}$.

14 $$\begin{aligned} C_l=C_{deg}+C_{cor} \end{aligned}$$

**Calculation of** $$C_{deg}$$:

The battery capacity loss also occurs due to the battery discharge cycle and is denoted as degradation capacity ($C_{deg}$). Normally, the nominal battery cycle is provided by manufacturers that 80% capacity is the maximum that can be used. However, the battery discharge cycle varies as per application. The degradation due to cycle and number of cycles is calculated in Eqs. (13) and (14).

15 $$\begin{aligned} C_{deg}\left( t \right) =C_{deg,m}e^{-c_{z}\left( 1-\frac{Z_{W}\left( t \right) }{1.6z_{0}} \right) } \end{aligned}$$

Where $C_{deg,m}$ is the maximum degradation capacity (80%), $c_z$ is constant (5), $Z_{W}$ is the number of cycle. Again, $Z_{W}$ is influenced by the state of charge impact ($f_{SOC}$(t)), battery discharge current ($I_{bdis}$) and acid stratification impact ($f_{acid}$).

16 $$\begin{aligned} Z_{W}\left( t \right) =\frac{1}{C_{N}}\int _{0}^{t}I_{bdis}\left( t \right) f_{SOC}\left( t \right) f_{acid}\left( t \right) dt \end{aligned}$$

Battery degradation capacity increases when the SOC of the battery decreases. the battery lifetime impact will be higher for longer low SOC from the last fully charged battery. This results in the loss of capacity with mechanical stress on the active mass. So, the function for the state of charge impact is denoted as $f_{SOC}$ which is calculated from the last full charge time to the present time.

17 $$\begin{aligned} f_{SOC}\left( t \right) =1+\left( C_{SOC_{0}} +C_{SOC_{mi}}\left( 1-SOC_{mi} \right) f_{I}\left( i,n \right) \left( t-t_{0} \right) \right) \end{aligned}$$

Where $c_{SOC,0}$ and $c_{SOC,min}$ represents when SOC is 0 and minimum respectively. $f_{I}(i,n)$ is the current influenced by sulfate crystal structure. The current influence factor is mainly due to the discharge current from a fully charged battery and is given in Eq. (15).

18 $$\begin{aligned} f_{I}\left( i,n \right) =\sqrt{\frac{C_{10}/10}{I_{b}\left( t \right) }}\root 3 \of {e^{\frac{n\left( t \right) }{3.6}}} \end{aligned}$$

$C_{10}$ is the charge capacity at 10H current. The number of bad charges is represented by *n* which increases from SOC 0.9 to 1 and is presented as:

19 $$\begin{aligned} n\left( t \right) =\frac{0.0025-\left( 0.95-SOC_{m} \right) ^{2}}{0.0025} \end{aligned}$$

The impact of acid stratification is represented as:

20 $$\begin{aligned} & f_{acid}\left( t \right) =1+f_{SF}\left( t \right) \sqrt{\frac{C_{10}/10}{\left| I_{b}\left( t \right) \right| }} \end{aligned}$$

21 $$\begin{aligned} & f_{SF}\left( t + \Delta t\right) =f_{SF}\left( t \right) +\left( f_{plus}\left( t \right) -f_{minus}\left( t \right) \right) \Delta t \end{aligned}$$

Where $f_{SF}$ is the degree of acid stratification, $f_{plus}$ and $f_{minus}$ are increase and decrease of acid stratification respectively.

**Calculation of** $$C_{cor}$$**:**

The corrosion layer adds resistance with internal battery resistance over the lifetime of the battery in which the corrosion layer grows in a positive electrode. The thickness of the layer ($\Delta W$) varies according to the corrosion voltage of the positive electrode. The loss of capacity is the proportional function of corrosion layer thickness. Therefore, the corrosion layer capacity is determined by Eq. (19).

22 $$\begin{aligned} C_{cor}\left( t \right) =C_{cor,m}\frac{\Delta W\left( t \right) }{\Delta W_{m}} \end{aligned}$$

Where $C_{cor,m}$ and $\Delta W_{m}$ are the maximum corrosion layer capacity and thickness respectively. Both the terms are calculated as:

23 $$\begin{aligned} & \Delta W\left( t \right) =\left\{ \begin{matrix} k_{s}\left( \left( \frac{\Delta W\left( t-\Delta t \right) }{k_{s}} \right) ^{1/1.6}+\Delta t \right) ^{0.6}, V_{cor}< 1.74\\ \Delta W\left( t-\Delta t \right) +k_{s}\Delta t, V_{cor}> 1.74 \end{matrix}\right. \end{aligned}$$

24 $$\begin{aligned} & \Delta W_{m}=L.k_{sm} \end{aligned}$$

## Framework of objective function

For a cost-effective and reliable microgrid system, this work has framed a multi-objective-based Grey wolf optimization algorithm. These objective functions are framed considering the economic aspect, and reliability aspect. However, as per the World Energy Trilemma (WET) index^45^, this work also evaluates the energy index feature. Hence, the objective function incorporates three-dimensional goals which are characterized as follows.

### Economic aspect

As per the economic aspect, the only concern is to minimize the index levelized cost of Energy (LCOE). This aggregates capital investment, operation, and maintenance costs of all the distributed energy resources. So, the objective function is:

25 $$\begin{aligned} min_{DV_1,DV_2} LCOE \end{aligned}$$

Where $DV_1$ and $DV_2$ are two decision variables. $DV_1$=${n_{wt}, n{PV}, n_{b}}$ and $DV_2$=*SOC* The LCOE is the derivation of total annual cost which includes capital, operation and maintenance (OM) cost and all sources dispatched power ($P_{dis}$). The total cost of WPG, SPG, BSS aggregates to the economic term $C_t$.

26 $$\begin{aligned} LCOE=\frac{\sum _{0}^{8760} C_t(C_W,C_P,C_b)}{\sum _{0}^{8760}P_{dis}} \end{aligned}$$

Again, $C_t$ depends on capital recovery factor (RF), So, total capital cost (TCC) is:

27 $$\begin{aligned} TCC=C_t\times RF \end{aligned}$$

where *RF* is recovery factor.

### Reliability aspect

The power generation from DERs with microgrids is highly affected due to the unforeseeable nature of RESs.The reliability indicator i.e. loss of power supply probability (LPSP) is adopted which is the shortage of power supply that doesn’t meet the load demand.

28 $$\begin{aligned} LPSP=\frac{\sum _{0}^{8760}P_{loss}\left( t \right) }{\sum _{0}^{8760}P_{L}\left( t \right) } \end{aligned}$$

### Energy index aspect

When generation power exceeds the load demand, the excess energy can be sold to the utility grid. However, for remote locations where the grid is not accessible, this energy will be dumped which violates the WTE criteria. So, the energy index term is calculated to minimize the excess power (EP) which are not served.

29 $$\begin{aligned} EP=\sum _{0}^{8760}\left( P_{L}\left( t \right) -P_{DER}\left( t \right) \right) \end{aligned}$$

Where $P_{DER}\left( t \right)$ = $P_{W}(t)$+$P_{PV}(t)$ $\pm$ $P_{b}(t)$ The energy index (EI) is:

30 $$\begin{aligned} EI=1-\frac{EP}{\sum _{0}^{8760}P_{L}} \end{aligned}$$

## Optimization of microgrid

For reliable and cost-effective microgrid operation, the aim is to choose the optimum size of a wind turbine, PV panel, and battery. In this, LCOE, LPSP, and EI are framed as three objective functions. To solve these complex structures of microgrids with stochastic climatic conditions, a meta-heuristic multi-objective optimization algorithm is proposed for Wind-Solar-Battery-based microgrids. This algorithm mainly determines the mathematical model for the social hierarchy of wolf^46^. It has three best solutions in which the fittest one is named $\alpha$, second and third bests are $\beta$ and $\delta$ respectively. The remaining solutions are named as $\omega$. The search boundaries are defined as a vector, $\vec {B}$=[$A_{w}, {{A_{PV}, B_{d}}}$ ] in which $A_{w}$, $A_{PV}$ and $B_{d}$ are the defined maximum area for PV panel, wind and allowable days for battery respectively.

**Fig. 2:**
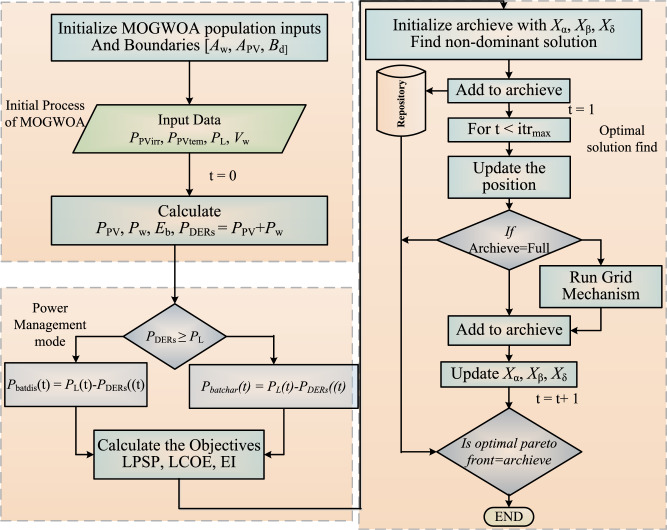
Flowchart of MOGWOA.

The flowchart of MOGWOA is presented in Fig. 2 and comprehensive analysis is done in MATLAB. To get the optimum solution, the following equations are used for implementation^46^.

31 $$\begin{aligned} & \overrightarrow{D}=\left| \overrightarrow{C} \overrightarrow{X_{p}}(i)-\overrightarrow{X}(i) \right| \end{aligned}$$

32 $$\begin{aligned} & \overrightarrow{X}(i+1)=\overrightarrow{X}_{p}(i)-\overrightarrow{A}\overrightarrow{D} \end{aligned}$$

33 $$\begin{aligned} & \overrightarrow{A}=2\overrightarrow{a}r_{1}-\overrightarrow{a}, \overrightarrow{C}=2r_{2} \end{aligned}$$

Where i: iteration number, $\overrightarrow{A}$ &$\overrightarrow{C}$: Co-efficient vector, $\overrightarrow{X_p}$ &$\overrightarrow{X}$: Position vector of prey and grey wolf respectively, $\overrightarrow{a}$: varies from 2 to 0, $r_1$, $r_2$: [0,1]: random number.

Further, for the three best solutions, the distance is calculated by using Eqs. (34–37).

34 $$\begin{aligned} & \overrightarrow{D_{\alpha }}=\left| \overrightarrow{C_1} \overrightarrow{X_{\alpha }}-\overrightarrow{X} \right| , \overrightarrow{X_1}=\overrightarrow{X_\alpha }-\overrightarrow{A}\overrightarrow{D_\alpha } \end{aligned}$$

35 $$\begin{aligned} & \overrightarrow{D_{\beta }}=\left| \overrightarrow{C} \overrightarrow{X_{\beta }}-\overrightarrow{X} \right| , \overrightarrow{X_2}=\overrightarrow{X_\beta }-\overrightarrow{A}\overrightarrow{D_\beta } \end{aligned}$$

36 $$\begin{aligned} & \overrightarrow{D_{\delta }}=\left| \overrightarrow{C} \overrightarrow{X_{\delta }}-\overrightarrow{X} \right| , \overrightarrow{X_3}=\overrightarrow{X_\delta }-\overrightarrow{A}\overrightarrow{D_\delta } \end{aligned}$$

37 $$\begin{aligned} & X\left( i+1 \right) =\frac{\overrightarrow{X}_{1}+\overrightarrow{X}_{2}+\overrightarrow{X}_{3}}{3} \end{aligned}$$

**Fig. 3:**
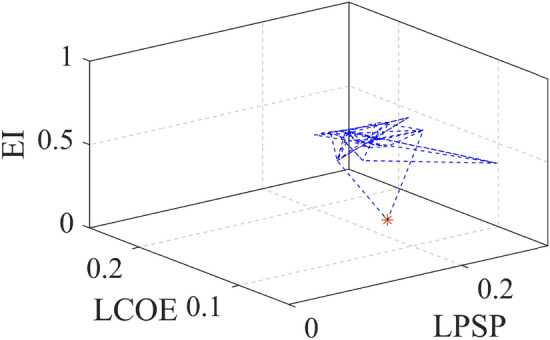
Pareto front of optimal solution.

This algorithm mainly contains three segments. The first one is the initial process. In this, the population size is 50 with 500 iterations. All the input data such as wind speed, solar irradiance, atmospheric temperature, and load data for one year are loaded. Then, the wind energy generation system, solar energy generation system, and battery energy storage system are modeled. From that, the wind power ($P_w$), solar power ($P_{PV}$) and battery energy ($E_b$), total DERs ($P_{DERs}$) total DERs generation is calculated.

### Analysis of optimal solution with performance measure

Here, the Pareto approach is used as the proposed method is also multi-objective optimization, where solutions are evaluated based on trade-offs between competing objectives. Instead of seeking a single optimal solution, the Pareto method identifies a set of solutions that represent the best possible compromises among the objectives, helping decision makers select the most suitable option based on their priorities. All the pareto solution with three-dimensional objective functions are obtained and shown in Fig. 3. The optimal point is converging as shown in one point. All the pareto solution with three dimensional objective functions are obtained and shown in Fig. 3. The optimal point is converging as shown in one point.

The Euclidean distance based approached is considered by calculating the distance between each solution to origin. The distance is given in Eq. (38).

38 $$\begin{aligned} D_{k}=\sqrt{(a-a_{k})+(b-b_{k})+(c-c_{k}))} \end{aligned}$$

Where $D_{k}$ is the distance between the origin and the non-dominant solution, *a*, *b*, *c* are the origin point, $a_{k}$, $b_{k}$, $c_{k}$ are the non-dominant Pareto font solution, min $D_{k}$ is chosen considering k = 1,2,..., *l* and *l* is the length of the archive.

The performance measure of MOGOW is measured by inverted generational distance (IGD) and metric of spacing (SP)? which is given in Eqs. (39) and (40) respectively. These parameter measures quality of the exploitation and exploration from pareto font.

39 $$\begin{aligned} IGD= & (\frac{1}{N}\sum _{i =1}^{N}\textrm{d}_{i}^{p})^{1/p} \end{aligned}$$

40 $$\begin{aligned} SP= & ((\frac{1}{N-1})\sum _{i = 1}^{N}(d_{av}-d_{m})^{p}))^{1/p} \end{aligned}$$

Where *N* is pareto solution number, $d_{i}$ is the Euclidean distance between $i_{th}$ pareto solution and each solution, $d_{av}$ is the average of distance.

This optimal solution of MOGWOA is compared with multi objective particle swan optimization (MOPSO) by considering the statistical min, max, average and standard values which is shown in Table 1.

**Table 1:** Performance comparison of MOPSO and MOGWO.

Statistical Parameter	Performance Parameter			
	MOPSO		MOGWOA	
	IGD	SP	IGD	SP
min	0.0107	0.0143	0.0029	0.0092
max	0.0149	0.123	0.0098	0.076
avg	0.0097	0.0216	0.004	0.0206
std	0.0221	0.0923	0.0022	0.022

## Results and discussion

**Fig. 4:**
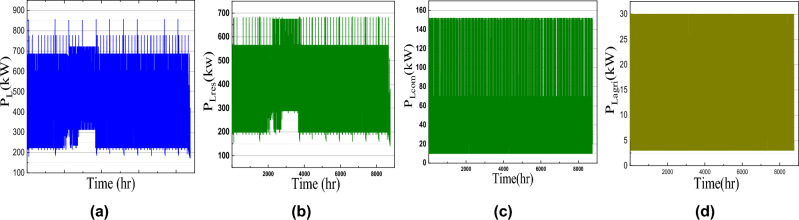
Load profile for a microgrid (**a**) Total load, (**b**) residential load, (**c**) commercial load and (**d**) agricultural load.

In this article, the load profile of one residential-based microgrid for Chennai, India location is considered for the MOGWOA optimization algorithm. This load consists of three different profiles i.e. typical residential load, commercial load, and agricultural load as shown in Fig. 4. As the load is situated in the subtropical region of South Asia, the load demand increases during summer. For day demand analysis, it is seen that for the forenoon from 6 AM to 10 AM, the load demand is higher than afternoon. Again in the evening after 6 AM, the light load increases the load demand. In this way, residential load demand is distributed for one year.

Commercial load demand remains high during the working hours of the day i.e. from 9 AM-6 AM and on Sunday the commercial load demand becomes half of the daily demand. Considering the agricultural load, the load demand is almost constant as shown in Fig. 4d. The annual solar irradiance, atmospheric temperature, and wind speed data are collected from PVGIS, EU as shown in Fig. 4. The battery parameter, wind turbine, PV panel, and cost model parameters are given in Table 1.

It is noted that the MOGOW optimization algorithm is used for three sets of configurations such as PV-battery, Wind-battery, and wind-pv-battery system, and found that the solution for the wind-pv-battery system is more reliable configuration for the particular load profile. For clarifications of the optimized results, the input cost model parameter is considered as given in Table 2. The optimum system configurations with various objective values are given in Table 3. The first column represents the input boundaries for the particular configuration and the second column is the optimized parameter rating. Considering the LCOE, the PV battery type configuration achieved less as compared to others. However, for the objective function, LPSP, only wind or PV with battery fails to meet the reliability indices. Hence wind-pv-battery-based configuration is more reliable. The energy index is also less in case III.

**Table 2:** Cost model parameter for optimized configurations.

Parameters	PV	Wind	Battery	Converter
Capital cost ($/kW)	650	1400	130	100
Maintenance cost ($/kW)	33	50	10	NA
Replacement cost ($/kW)	NA	NA	200	150
Lifespan (year)	25	25	5	15

Please add the following required packages to your document preamble:

**Table 3:** Optimized results for different configurations.

Configuratons	Boundaries			System Capacities			LCOE ($/kW)	LPSP	EI	ALCE ($)
	Wind($\hbox {m}^2$)	PV($\hbox {m}^2$)	Battery (days)	Wind (kW)	PV (kW)	Battery (kWh)				
Wind-Battery	(5000–20000)	–	1–3	2516	–	17810	0.2	0.26	0.3	906790
PV-Battery	–	2000–18000	1–3	–	2474.162	16605	0.1025	0.19	0.2	477550
Wind-PV-Battery	4,000–12,000	1500–7,000	1–3	1728.7	885	28648	**0.1011**	**0.173**	**0.1858**	**821160**

Significant values are in bold.

**Fig. 5:**
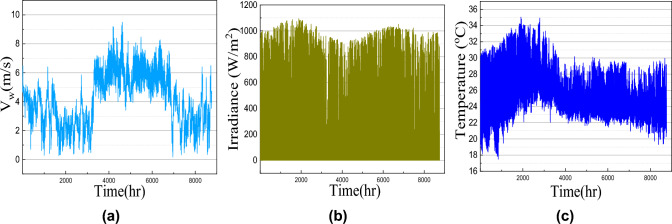
Climatic Profile (**a**) Wind speed, (**b**) Solar irradiance and (**c**) Temperature profile.

**Fig. 6:**
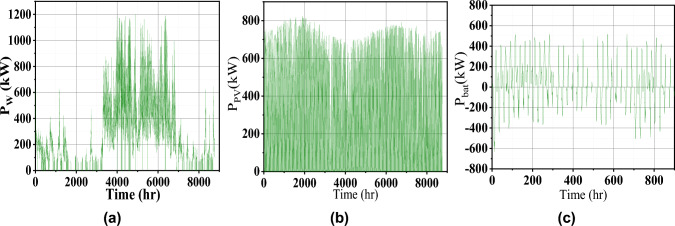
Microgrid operation characteristics (**a**) generated wind power, (**b**) PV power, and (**c**) battery power.

**Fig. 7:**
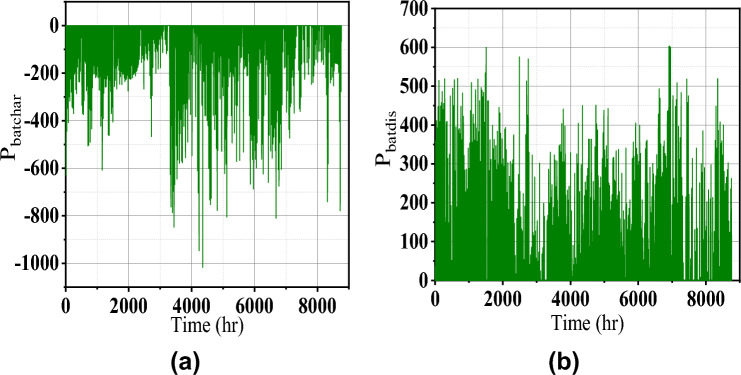
Battery charge and discharge power.

**Fig. 8:**
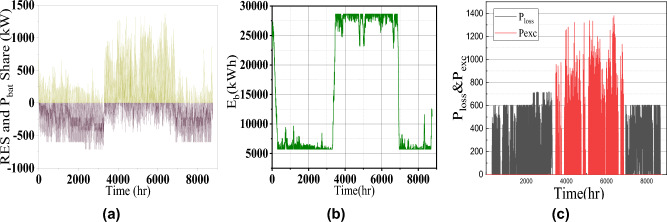
Microgrid operation characteristics (**a**) Cumulative DER power and battery power, (**b**) Battery energy and (**c**) excess and shortage power.

**Fig. 9:**
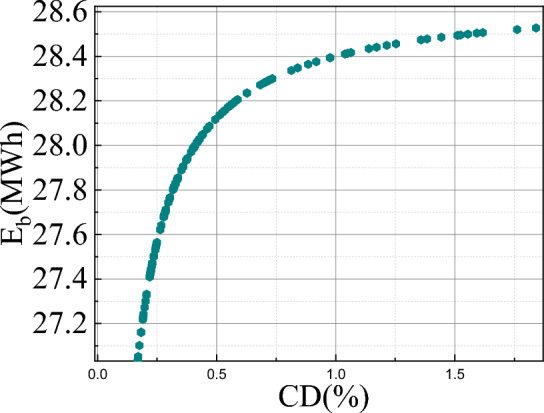
Annual capacity degradation of battery.

For more visualization of the results, the 3D Pareto front (Fig. 3) is analyzed in which the variable parameters are LCOE, LPSP, and EI to meet the sustainable energy requirement. Table 1 presents the design and performance metrics for three configurations. It is noted that the 1728.7kW of Wind energy generation system, 885kW of PV, and 28648kWh of battery is the optimal size for this given microgrid system. The generated wind, PV, and battery power for one one-year time scale are shown in Fig. 5. The average RESs share with battery contribution for the high load period in May month is shown in Fig. 6a which confirms the sustainable energy management within the microgrid (Fig. 7).

During summer, the residential and agricultural load increases due to excess demand. Moreover, PV power is more in the daytime, and wind power complements the PV power at night. So the power balance is maintained as shown in Fig. 8. The corresponding charge and discharge power of the battery is shown in Fig. 7 which again reveals the satisfactory energy management.

Considering the energy index of the system, loss of power and excess energy are complementary to each other. It is seen that during the second quarter of the year, 28% of energy can be fed back to the utility grid or can be sold to other nearby grids which generate revenue. The energy index of the proposed system configuration is 0.1858 which indicates less surplus energy.

Battery degradation factor (CD: capacity degradation & $E_b$: battery capacity) is shown in Fig. 9 concerning the battery capacity for one year. Battery capacity has a high impact on the charge and discharge cycle of the system. It is seen that the increase in battery energy with depth of discharge of the battery leads to an increase in capacity degradation factor (1.75). This is due to the sulfate crystal size increase and also the increase of mechanical stress on the active mass (as given in Eqs. 10–21). Battery lifetime calculated for the wind-PV-battery-based microgrid system is 12.5 years from the annual capacity factor considering 80% of DOD.

All three optimized configuration results are summarized in Table 2. It is noted that the last hybrid configuration which is wind-PV-battery meets the desired design criteria i.e. more reliability (LPSP= 0.173) and less excess energy with reduced LCOE (0.1011$/kw). However, battery capacity is higher than the two other configurations because of the highly fluctuating nature of wind speed. Moreover, for the PV-battery configuration, LCOE and battery capacity are comparatively lower than the wind-battery one.

## Conclusion

In this article, a multi-objective GWO algorithm is proposed for a wind-PV-battery-assisted microgrid model. The global search capability of this algorithm converges to the optimal solution and meets the three-dimensional WET goal. A three-dimensional objective function considering LCOE, LPSP, and EI is developed to meet the design criteria of the microgrid. Therefore, this system delivers reliable and sustainable power to remotely located microgrids with reduced surplus energy which is indicated by the energy index term. In addition, microgrid operation is described by designing one detailed model for all DERs by taking into account all the constraints. Moreover, computational studies are included for the detailed lead-acid battery degradation model to calculate the battery replacement during the system lifetime which also enhances the system reliability. The proposed configuration achieves affordable per unit cost of electricity (0.12011 $/kW) with high reliability (82%) and less EI (0.1858). All the microgrid operation results with the proposed configuration are evidence for the feasibility of the MOGWO algorithm.

## Data Availability

The datasets used and/or analyzed during the current study are available from the corresponding author upon reasonable request.
